# Temoporfin-in-Cyclodextrin-in-Liposome—A New Approach for Anticancer Drug Delivery: The Optimization of Composition

**DOI:** 10.3390/nano8100847

**Published:** 2018-10-18

**Authors:** Ilya Yakavets, Henri-Pierre Lassalle, Dietrich Scheglmann, Arno Wiehe, Vladimir Zorin, Lina Bezdetnaya

**Affiliations:** 1 Centre de Recherche en Automatique de Nancy, Centre National de la Recherche Scientifique UMR 7039, Université de Lorraine, Campus Sciences, Boulevard des Aiguillette, 54506 Vandoeuvre-lès-Nancy, France; i.yakavets@nancy.unicancer.fr (I.Y.); h.lassalle@nancy.unicancer.fr (H.-P.L.); 2 Research Department, Institut de Cancérologie de Lorraine, 6 Avenue de Bourgogne, 54519 Vandoeuvre-lès-Nancy, France; 3 Laboratory of Biophysics and Biotechnology, Belarusian State University, 4 Nezavisimosti Avenue, 220030 Minsk, Belarus; vpzorin@mail.ru; 4 Biolitec Research GmbH, Otto-Schott-Strasse 15, 07745 Jena, Germany; dietrich.scheglmann@biolitec.com (D.S.); arno.wiehe@biolitec.com (A.W.); 5 International Sakharov Environmental Institute, Belarusian State University, Dauhabrodskaja 23, 220030 Minsk, Belarus

**Keywords:** temoporfin, drug-in-cyclodextrin-in-liposome, nanoparticles, multicellular tumor spheroids, flow cytometry, photodynamic therapy

## Abstract

The main goal of this study was to use hybrid delivery system for effective transportation of temoporfin (*meta*-tetrakis(3-hydroxyphenyl)chlorin, mTHPC) to target tissue. We suggested to couple two independent delivery systems (liposomes and inclusion complexes) to achieve drug-in-cyclodextrin-in-liposome (DCL) nanoconstructs. We further optimized the composition of DCLs, aiming to alter in a more favorable way a distribution of temoporfin in tumor tissue. We have prepared DCLs with different compositions varying the concentration of mTHPC and the type of β-cyclodextrin (β-CD) derivatives (Hydroxypropyl-, Methyl- and Trimethyl-β-CD). DCLs were prepared by thin-hydration technique and mTHPC/β-CD complexes were added at hydration step. The size was about 135 nm with the surface charge of (−38 mV). We have demonstrated that DCLs are stable and almost all mTHPC is bound to β-CDs in the inner aqueous liposome core. Among all tested DCLs, trimethyl-β-CD-based DCL demonstrated a homogenous accumulation of mTHPC across tumor spheroid volume, thus supposing optimal mTHPC distribution.

## 1. Introduction

Application of nanomaterials in drug delivery of anticancer therapeutics has become one of the greatest challenges in cancer therapy, including photodynamic therapy (PDT) [1]. PDT is based on the combined action of photosensitizer (PS), light, and molecular oxygen, leading to the generation of toxic reactive oxygen species, which in turn can damage tumor tissue [2,3]. High accumulation and penetration of PS are primary factors that are responsible for PDT efficiency due to the high selectivity of its treatment. The main cytotoxic species, the singlet oxygen (^1^O_2_), has only a short lifetime in biological media and consequently limited action distance, therefore non-homogeneous distribution of PS into the tumor mass may lead to incomplete tumor eradication [4]. In turn, nanotechnology-based drug delivery systems could significantly improve the transportation of poorly soluble PS providing the opportunities for active targeting, controlled release, and multimodality. Nonetheless, all developed nanomaterials are not without drawbacks, such as quick uncontrolled release of the drug, rapid clearance, limited penetration in tissues, etc. [5]. Therefore, hybrid systems as a combination of several nanomaterials have been recently suggested as an alternative approach. The present work deals with the application of hybrid delivery system based on the combination of liposomes and cyclodextrins for the clinically approved PS temoporfin.

**Scheme 1:**
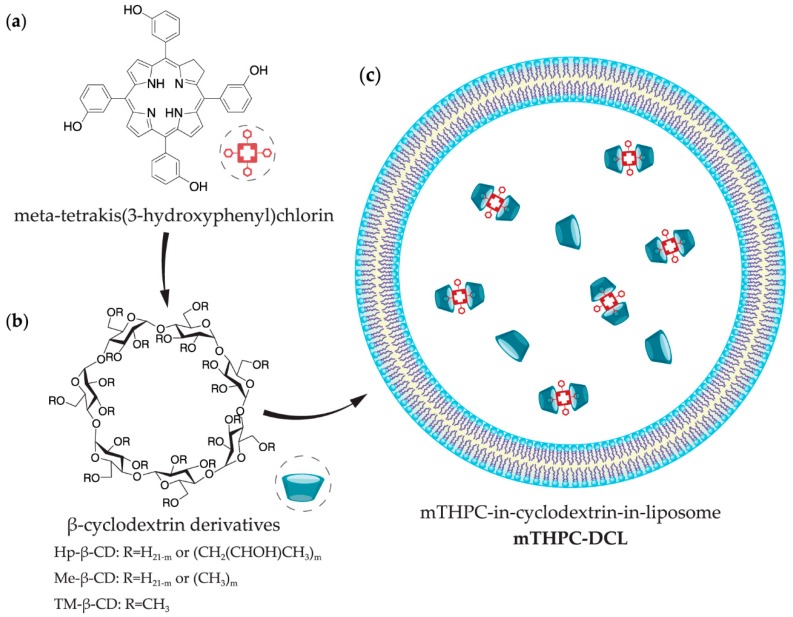
Representative scheme of (**a**) *meta*-tetrakis(3-hydroxyphenyl)chlorin (mTHPC), (**b**) β-cyclodextrin (β-CD) derivatives, and (**c**) drug-in-cyclodextrin-in-liposome (DCL) nanoconstruct.

Temoporfin (*meta*-tetrakis(3-hydroxyphenyl)chlorin, mTHPC) is a highly potent second-generation photosensitizer (Scheme 1a). The commercial medicinal product that is based on mTHPC (Foscan^®^) requires low light doses and concentrations to be photoactive and has already been approved by the European Medicines Agency (EMA) for the palliative treatment of advanced head and neck squamous cell carcinoma since 2001 [6,7]. Recently, we demonstrated a unique possibility to alter the biodistribution of mTHPC using β-cyclodextrin (β-CD) derivatives (Scheme 1b) [8,9]. The binding constants of mTHPC with β-CD derivatives vary from 10^5^ to 10^7^ M^−1^ [10], providing the unique “nanoshuttle” transportation mechanism of mTHPC in biological media. Depending on the concentration of β-CD derivatives, they could either accelerate mTHPC distribution from serum proteins to tumor targets increasing cellular uptake and providing a deeper mTHPC penetration in three-dimensional (3D) tumor models or “isolate” drug molecules hampering PS delivery [9]. Such remarkable results in vitro strongly depend on the local concentration of β-CD, and as such, in vivo application of β-CD formulations is complicated due to the quick β-CD removal from the circulating system after intravenous injection [8]. The control release of inclusion complexes in target tissue may be better achieved in the case of hybrid drug-in-cyclodextrin-in-liposome (DCL) formulation (Scheme 1c).

DCL nanoconstruct, which was firstly proposed by McCormack and Gregoriadis (1994) [11], was already used for the delivery of a number of lipophilic bioactive molecules, including anti-inflammatory, anesthetic, immunosuppressive, and anti-cancer drugs [12,13]. Based on that, we suppose that the encapsulation of β-CD-complexed mTHPC into liposomes increases the drug loading capacity, entrapment efficiency, avoids burst release of the drug, and prolongs its systemic circulation. The resultant DCL could restrain the dissociation of drug-mTHPC complexes, avoid rapid drug release, and contribute to the altering PSs pharmacokinetics in vivo. To the best of our knowledge, such nanoconstruct, associating PS/β-CD complexes and liposomes has never been proposed before. Thus, the comprehensive optimization of DCL composition is needed to achieve appropriate mTHPC intra-tissue distribution. 

The present work is focused on the optimization of DCL composition. With this aim, we varied mTHPC concentration and the type of β-CD to achieve the most appropriate penetration of PS into the multicellular tumor spheroids. Additionally, we characterized DCLs spectroscopically and studied their colloidal stability.

## 2. Materials and Methods

### 2.1. Materials

Liposomal mTHPC formulation, Foslip^®^, was kindly provided by biolitec research GmbH (Jena, Germany). mTHPC stock solution was prepared in methanol and kept at 4 °C in the dark. The concentration of mTHPC in the solution was estimated spectroscopically using molar extinction coefficient of 30,000 M^−1^ cm^−1^ at 650 nm in ethanol [14]. Foslip^®^ is based on L-a-dipalmitoylphosphatidyl-choline (DPPC), dipalmitoylphosphatidylglycerol (DPPG), and mTHPC, with a drug:lipid ratio of 1:12 (mol/mol) and a DPPC:DPPG ratio of 9:1 (*w*/*w*). Foslip^®^ was reconstituted from lyophilized powder in distilled water as per the manufacturer’s instructions.

DPPC and DPPG were purchased from (Sigma-Aldrich, St. Louis, MO, USA). 2-hydroxypropyl-β-cyclodextrin (Hp-β-CD; product code CY-2005.2,27; substitution degree of 4.5, average molecular weight 1135 Da), random methyl-β-cyclodextrin (Me-β-CD; product code CY-2004.1,29; substitution degree of 12, average molecular weight 1135 Da), and heptakis(2,3,6-tri-*O*-methyl)-β-cyclodextrin (TM-β-CD; product code CY-2003,34; molecular weight 1429.6 Da) were purchased from CYCLOLAB R&D. Ltd., (Budapest, Hungary). For in vitro cell culture experiments, we used phenol red-free Roswell Park Memorial Institute 1640 medium (RPMI-1640, Invitrogen™, Carlsbad, CA, USA), supplemented with 2% fetal bovine serum (FBS) (Life Technologies, Carlsbad, CA, USA). Ultrapure water (RiOs™ 8, Millipore, Milli-Q^®^ Advantage A10^®^ System, Millipore, Eschborn, Germany) was used for sample preparation.

### 2.2. Drug-in-Cyclodextrin-in-Liposome Vesicles Preparation

#### 2.2.1. Preparation of Inclusion Complexes

Inclusion complexes between β-CDs and mTHPC were formed using co-evaporation method [15]. Briefly, β-CDs were dissolved in ultrapure water (UPW) at the required concentrations. mTHPC stock solution (10 mM) was prepared in methanol. mTHPC was afterwards added to β-CDs solution (1:2 *w*/*w*) and solvent was removed by rotary evaporation (Rotavapor R-100, Büchi Labortechnik AG, Flawil, Switzerland) at 65 °C. mTHPC/β-CDs complexes were dissolved in UPW at the required concentrations and were kept at 4 °C in the dark.

#### 2.2.2. Thin Film Hydration

The composition of the prepared different liposomal formulations is provided in Table 1. Unilamellar liposomes were made by filter extrusion technique. All liposomal vesicles contained a combination DPPC and DPPG at a molar ratio 9:1. The total amount of lipids was 20 mg/mL (26 mM) for all formulations. Empty liposomes were prepared by thin lipid film hydration followed by extrusion, as was described previously [16]. Briefly, 18 mg/mL of DPPC and 2 mg/mL of DPPG were dissolved in 0.6 mL of 99.6% ethanol. A thin film was obtained by the removal of the solvent by rotary evaporation at 65 °C. The film was hydrated in 0.3 mL of UPW and underwent three freeze-thaw cycles. The suspension was extruded 21 times through 100 nm polycarbonate Nuclepore^®^ membranes using Avanti Mini-Extruder (Avanti, Alabaster, AL, USA) at 60 °C. After extrusion liposomes were stored at 4 °C. Liposomes containing mTHPC/β-CDs complexes (DCLs) were prepared in the same manner. The mTHPC/β-CDs complexes were added at the lipid film hydration step, as was previously reported [17].

Throughout the paper, abbreviations HDCL, MDCL, and TDCL stand for DCL encapsulated mTHPC complexes with various β-CD derivatives (Hydroxypropyl-, Methyl-, and Trimethyl-β-CD), with each of them containing three different mTHPC concentrations (5 mM, 1.7 mM, and 0.5 mM, denoted as 1–3, respectively) (Table 1).

#### 2.2.3. Purification of DCLs

To purify DCL samples from not encapsulated mTHPC/β-CDs, a minicolumn chromatography technique was used [18]. Briefly, minicolumns from 2.5 mL syringes pre-filled with Sephadex G-50 superfine (GE Healthcare Life Sciences, Buckinghamshire, UK) were dried by centrifugation for 3 min at 2000 *g*. Then, the DCL samples were placed into the minicolumns and were collected after centrifugation at the same conditions. 

### 2.3. Characterization of Liposomes

#### 2.3.1. Determination of Encapsulation Efficiency (EE)

Encapsulation efficiency of mTHPC in DCLs was calculated as the ratio (*A*_final_/*A*_total_ × 100%), where (*A*_total_) is mTHPC optical density measured immediately after extrusion and *A*_final_ is the mTHPC optical density measured after purification. The optical density was measured at 652 nm for the samples that were diluted 1500 times in 0.2% Triton^®^ X-100 solution.

#### 2.3.2. Photon Correlation Spectroscopy (PCS)

Particle size (hydrodynamic diameter) and polydispersity index (PDI) were determined by PCS (Zetasizer Nano ZS, Malvern Instruments, Worcestershire, UK). The samples were diluted 1000 times in ultrapure water at 25 °C. The measurements were performed at the angle of 173°. The calculations of particle size were done assuming a spherical particle shape, a medium viscosity of 0.89 mPas, and a refractive index of 1.33. The results were presented as the mean of three consecutive measurements. 

#### 2.3.3. Atomic Force Microscopy (AFM)

Imaging was performed using an atomic force microscope Solver P47PRO (NT-MDT, Moscow, Russia). Images were done in semi-contact scanning mode at the frequency 150 kHz using NSG 11 cantilever. The ROTH slides (Carl Roth GmbH, Karlsruhe, Germany) with the films of DCLs samples being diluted 10 times were dried on the air for 10–12 h before the experiment. Processing and analysis of images were carried out using the offline portion of NT-MDT Image Analysis software (software version 2.2).

#### 2.3.4. Spectroscopic Measurements

Absorption measurements were recorded with a Lambda 35 spectrometer (PerkinElmer, Waltham, MA, USA) using 1 cm optical path quartz cuvettes. Fluorescence measurements were conducted with LS55B spectrofluorometer (PerkinElmer, Waltham, MA, USA) equipped with polarizers, thermostated cuvette compartments, and magnetic stirring. Induced circular dichroism spectra were registered by Chirascan-plus qCD (Applied Photophysics Limited, Surrey, UK), which was equipped with thermostat. All spectroscopic measurements were carried out at a room temperature (23–25 °C). Optical density of all samples did not exceed 0.4 a.u. All of the measurements were performed in triplicate.

### 2.4. Monolayer and Spheroid Cell Cultures

#### 2.4.1. Culture Conditions

Human colon adenocarcinoma cells HT29 were obtained from the ATCC^®^ (LGC Promochem, Molsheim, France) and controlled for mycoplasma contamination. Cells were maintained in RPMI-1640 medium (Thermo Fisher Scientific, Waltham, MA, USA) supplemented with 9% (*v*/*v*) heat-inactivated fetal calf serum, antibiotics (penicillin 10,000 IU and streptomycin 10,000 μg/mL), and 1% (*v*/*v*) 0.1 M glutamine (Life Technologies, Carlsbad, CA, USA). Cells were kept as a monolayer culture in a humidified incubator (5% CO_2_) at 37 °C. 

#### 2.4.2. Generation of Spheroids

The procedure of initiation of multicellular tumor spheroids (MCTS) by spinner technique was described previously [9,19]. Cell aggregates were transferred to 250 mL spinner flasks (Integra Biosciences, Zizers, Switzerland) containing 150 mL of culture medium. The spinner flasks were then placed on magnetic plates (Integra Biosciences) at 75 rpm in 5% CO_2_ and 37 °C humidified atmosphere. The culture medium was changed every 2–3 days. MCTSs were used for experiments, once they reached 500 µm in diameter (after 15 days).

In order to dissociate MCTSs, they were transferred into a flask (15 mL), then washed twice with PBS, and further incubated with 0.05% trypsin (GIBCO™, Thermo Fisher Scientific, Waltham, MA, USA), 0.02% ethylenediaminetetraacetic acid (EDTA, GIBCO™, Thermo Fisher Scientific). Afterwards, the complete culture medium was added to inhibit trypsination. Finally, cell suspension was centrifuged to a pellet and resuspended in fresh culture medium.

#### 2.4.3. Imaging of mTHPC Distribution

HT29 cells (3 × 10^4^ cells/mL) were plated into Lab-Tek II chamber Slide (Roskilde, Denmark), incubated in the dark at 37 °C with 4.5 μM of mTHPC in different formulations for 3 h, and then rinsed with PBS. The experiments on MCTSs with mTHPC formulations were performed in accordance with the previously published protocol [9]. Briefly, DLCs and mTHPC liposomes were added to the samples for 24 h. The concentration of mTHPC was 4.5 μM. The incubation of samples was performed in the dark conditions in a humidified incubator (5% CO_2_) at 37 °C. Afterwards, MCTSs have been transferred to Petri dishes, then washed twice with PBS, and finally directly observed by epifluorescence microscope.

mTHPC fluorescence was observed under an upright epifluorescence microscope (AX-70 Provis, Olympus, France). The filter was se at 400–440 nm band pass excitation associated with a 570 nm dichroic mirror and a 590 nm long pass emission filter for mTHPC fluorescence measurements. Fluorescence images were recorded using an oil immersion ×40 objective in the case of cellular monolayer and ×4 objective for spheroid cell culture. The line profiles of mTHPC fluorescence across spheroids were calculated using ImageJ software from 10 radial lines, which were randomly drawn at the spheroid images.

### 2.5. Statistical Analysis

Student’s *t*-test was used for the statistical analysis. Statistical significance was established at *P* < 0.05. All experiments were repeated at least three times and the results were expressed as mean values ± S.D.

## 3. Results and Discussion

### 3.1. Characterization of mTHPC-DCLs

#### 3.1.1. Preparation of mTHPC-DCLs

mTHPC-loaded DCLs were prepared by thin hydration method with various β-CD derivatives containing different amounts of mTHPC. In practice, the soluble mTHPC/β-CD inclusion complexes were encapsulated in an aqueous core of conventional liposomes at the step of lipid film hydration, thus forming DCLs. Such methodology is considered as the most widely used method for DCLs preparation [17,20,21,22]. After the reduction of the size of lipid vesicles by extrusion, mTHPC/β-CD loaded liposomes were purified by means of minicolumn chromatography technique to eliminate external PS/cyclodextrin inclusion complexes [18]. This minicolumn chromatography allows for easy separation of small inclusion complexes from bulk liposomes with minor dilution in a short time (about 5 min). Other purification techniques like ultracentrifugation can damage the liposomes and stimulate their aggregation, while the dialysis technique is very slow (about 24 h) and unexpected mTHPC redistribution from liposomes to β-CD in medium can happen.

To optimize DCL composition, we varied mTHPC concentration as well as the type of β-CDs. To prepare the solutions of inclusion complexes we used mTHPC concentrations as 0.5, 1.7, and 5 mM. DCLs, prepared with these solutions, were named as DCL3, DCL2, and DCL1, respectively (Table 1). The concentration of β-CD was chosen in a way to completely prevent mTHPC aggregation at 5 mM (data not shown). The concentrations for Hp-, M-, and TM-β-CD were 200, 20, and 10 mM, respectively (HDCL, MDCL, and TDCL). The total lipid concentration was 26 mM for all DCLs in order to achieve high concentration of lipid vesicles and higher amount of encapsulated inclusion complexes in them.

According to the data obtained, EE of mTHPC in DCLs depends on mTHPC concentration added during DCL preparation (Table 2). The highest amount of mTHPC encapsulated in DCLs was observed for solution of inclusion complexes with 0.5 mM of mTHPC (DCL3) as compared to DCL2 and DCL1. However, EE did not exceed 20%, irrespective of mTHPC-DCL formulations. In contrast, the encapsulation of hydrophobic mTHPC molecules in lipid bilayer is characterized by the EE more than 85%, according to the literature data [16]. It is well known that EE values of soluble drugs incorporated into the inner aqueous liposomal core are strongly limited by drug solubilization and are much lower than the EE of hydrophobic molecules encapsulated into lipid bilayer. Evidently, low EE is a limiting factor for the potential commercial application of DCLs. However, this could be improved by the additional encapsulation of hydrophobic drugs into the lipid membrane of liposomes (double loaded DCL) [23].

#### 3.1.2. Size and Zeta Potential

**Figure 1:**
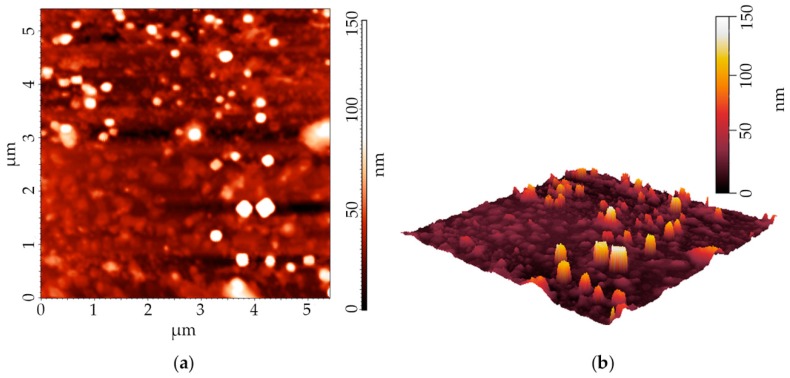
(**a**) Typical height image of DCL by means of atomic force microscopy. (**b**) Three-dimensional (3D) reconstruction of DCL height image. The presented image corresponds to the MDCL1.

Morphology and size of DCLs and Foslip^®^ were analyzed by means of atomic force microscopy and PCS. The physical characteristics of DCLs are summarized in Table 2. As shown in Figure 1a, the AFM images of DCLs illustrate a spherical morphology. The representative topography and three-dimensional (3D) reconstruction of AFM image is presented in Figure 1b. The analysis of the line profiles demonstrates the presence of spherical vesicles ≈110 nm with appropriate homogeneity. The hydrodynamic size and zeta-potential of the nanoparticles (NPs) were precisely analyzed by PCS (Table 2). In accordance with the previously reported data [24,25], Foslip^®^ solution exhibits homogenous distribution in lipid vesicles with the diameter of 113.6 ± 0.7 nm (PDI = 0.110 ± 0.015). Mean hydrodynamic diameter of DCLs varied between 125.7 ± 0.9 and 142.2 ± 0.8 nm. DCL populations were homogeneous in size (PDI varied between 0.037 ± 0.015 and 0.146 ± 0.040). Additionally, no obvious differences were detected in the mTHPC-DCLs particle sizes in function of the types of β-CDs.

Zeta-potential of NPs was also measured to characterize their surface charge. The lipid membrane of Foslip^®^ consists of negatively charged lipid in the membrane (DPPG) with a ratio 1:9 DPPG/DPPC. Thus, zeta-potential of Foslip^®^ is (−34.4 ± 4.3) mV. Similar composition of lipid membrane was used for DCL formulations; therefore all the studied DCLs had strong negative surface charge from (−36.7 mV) to (−39.0 mV). Obviously, negative charge of vesicles (<−30.0 mV) could prevent its aggregation in solution and determines their high colloidal stability [26,27]. Moreover, it should be taken into account that negative surface charge is also important in terms of NPs interactions with cells and could determine their efficacy in vivo [28,29].

#### 3.1.3. mTHPC Localization in DCLs

The localization of mTHPC in DCLs plays an important role in terms of understanding the delivery mechanism of mTHPC by hybrid nanoconstructs. It is acknowledged that mTHPC exhibits high affinity to the lipid environment [30]. Therefore, it would be hardly possible that mTHPC is completely bound with β-CDs in the inner aqueous core of DCLs. In all probability, mTHPC molecules partially redistribute between inclusion complexes and a liposomal bilayer. Actually, the localization of mTHPC in DCLs may be analyzed by spectroscopic techniques as a special case of equilibrium competitive binding [31,32].

**Figure 2:**
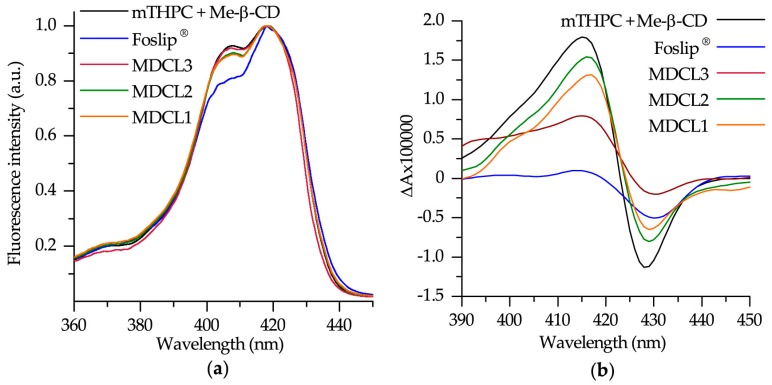
(**a**) Normalized fluorescence excitation spectra of 1 µM mTHPC in different formulations. Fluorescence signal was measured at 652 nm. (**b**) Induced circular dichroism spectra of 1 µM mTHPC in different formulations. Concentration of Me-β-CD was 0.2 mM.

The spectral data related to Foslip^®^, DCLs loaded with mTHPC/Me-β-CD inclusion complexes (MDCLs) and mTHPC/Me-β-CD inclusion complexes are presented in Figure 2. Spectral characteristics of mTHPC/Me-β-CD inclusion complexes and Foslip^®^ were previously described and were used here only for comparative analysis [16,32]. To estimate the fraction of mTHPC bound to β-CDs, we used two different spectral techniques: conventional circular dichroism and spectroscopic based approach related to the shape of Soret band (Figure 2) [31,32].

**Scheme 2:**
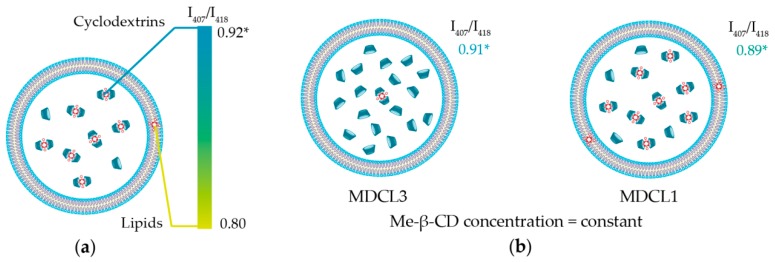
(**a**) Schematic representation of possible localization of mTHPC in MDCL. Color bar displays the range of *I*_407_/*I*_418_ ratios. Maximal value (0.92) corresponds to complete binding of mTHPC to Me-β-CD, while minimal ratio (0.80) displays the location of all mTHPC molecules in lipid bilayer. (**b**) Schematic representation of mTHPC localization in MDCLs depending on the initial mTHPC concentration. *—all values correspond to mTHPC/Me-β-CD inclusion complexes.

As seen in Figure 2a, the shape of mTHPC Soret band in DCLs is similar to that of mTHPC/Me-β-CD. According to our recent study [32], the shape of Soret band of mTHPC fluorescence excitation spectra is sensitive to the changes of mTHPC microenvironment and *B*_x_/*B*_y_ ratio could be used for the quantitative analysis. The mTHPC/Me-β-CD inclusion complexes are characterized by *I*_407_/*I*_418_ = 0.92 ratio (where *I*_407_ and *I*_418_ stand for the fluorescence intensities at 407 nm and 418 nm excitation respectively), while liposomes (Foslip^®^) exhibits *I*_407_/*I*_418_ = 0.80 (Scheme 2a). The calculated *B*_x_/*B*_y_ ratios for mTHPC in MDCLs are equal to 0.89, 0.90, and 0.91 for MDCL1, MDCL2, and MDCL3 respectively. It corresponds to the mTHPC fraction of 75%, 83% and 92% bound to Me-β-CD in the inner aqueous core (Scheme 2b). Similar tendency was observed for other types of β-CDs (data not shown). We obtained that for TM-β-CD the fraction of mTHPC in inclusion complexes for all TDCLs exceeds 90%. At the same time, the percentage of mTHPC bound to Hp-β-CD was 70%, 80%, and 90% for HDCL1, HDCL2, and HDCL3, respectively. The preferable localization of mTHPC in inclusion complexes was further confirmed by circular dichroism technique (Figure 2b). However, circular dichroism is an absorbance-based technique, therefore medium turbidity could also complicate the quantitative analysis, as was described already for absorbance spectra.

Summarizing this spectroscopic part, we can conclude that mTHPC molecules are mainly located in the inclusion complexes with β-CDs in the inner aqueous core of DCLs. We demonstrated that the localization of mTHPC strongly depends on the β-CDs’ affinity to mTHPC and concentration of β-CD what is in a good agreement with the competitive mechanism of mTHPC binding. For instance, binding constant of TM-β-CD with mTHPC is >10^7^ M^−1^ [10], which is at least ten times higher than these for other β-CDs. Such tight binding of PS in the inclusion complexes allows TDCLs encapsulate almost all mTHPC (>90%) in the inner aqueous core. On the contrary, decreasing of mTHPC concentration upon complex preparation leads to the increase of concentration of free β-CDs in the cavity (Scheme 2b). Therefore, in the case of DCL3, the amount of mTHPC bound in inclusion complexes is higher than for DCL1.

#### 3.1.4. Storage Stability

**Figure 3:**
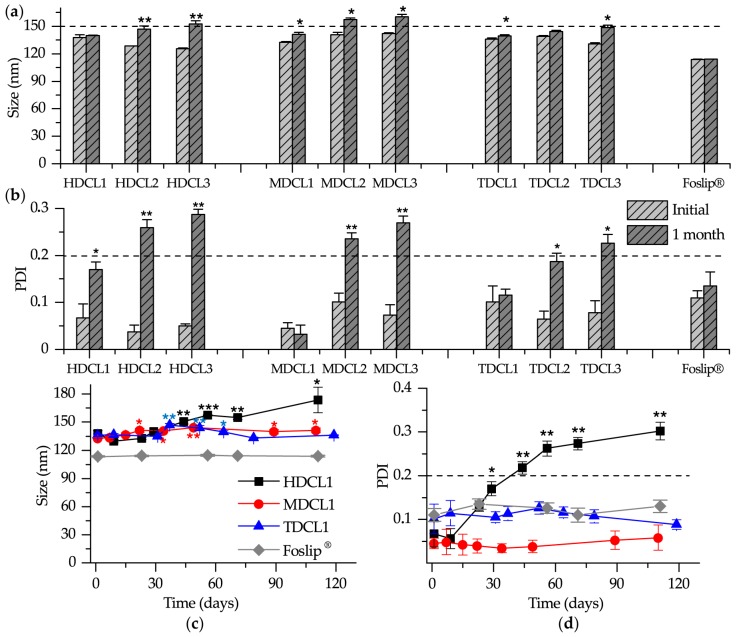
(**a**) Size and (**b**) polydispersity index (PDI) of mTHPC formulations after one month of the storage. Kinetics of (**c**) size and (**d**) PDI of HDCL1, MDCL1, TDCL1 and Foslip^®^ during three months of storage. The samples were stored at 4 °C in the dark. The samples were diluted 1000 times in ultrapure water (UPW). *—statistically different from initial sample, *P* < 0.05; **—statistically different from initial sample, *P* < 0.01, ***—statistically different from initial sample, *P* < 0.001.

It is acknowledged that β-CDs remove lipid components from liposomes, thus destabilizing them [33,34]. The stability of different liposome dispersions was investigated by means of PCS analysis during their storage at 4 °C in the dark (Figure 3). Foslip^®^ was used as a reference sample since according to the manufacturer it is stable during more than six months after dissolution. No marked variations in the size were observed for DCL1 (HDCL1, MDCL1 and TDCL1) after 1 month of storage (Figure 3a). The size of DCLs with low initial mTHPC loading (DCL2 and DCL3) was slightly increased, especially for HDCLs (Figure 3a). At the same time, the measurements of PDI clearly demonstrated that PDI for HDCL1, MDCL1, and TDCL1 were in the range of acceptable homogeneity (PDI < 0.2), while DCL2 and DCL3 for all β-CDs were characterized by PDI > 0.2 (Figure 3b). Meanwhile, the PDI of HDCL1 was significantly increased after 1 month and tended to degrade after 1.5 month (Figure 3c,d). Similarly to Foslip^®^, MDCL1 and TDCL1 exhibit the colloidal stability during more than three months.

Obviously, the observed heterogeneity of DCLs with low initial mTHPC loading (DCL2 and DCL3) could be due to β-CD-induced liposomes’ destabilization. Cyclodextrins are known to interact with lipid components removing them from membrane by forming inclusion complexes with lipids [30,35]. The intensity of such process is mainly determined by the relative concentration of free β-CD molecules [36]. According to that, when we decrease initial mTHPC concentration used for DCL preparation, the number of free β-CD molecules in the aqueous core increases accelerating destabilization of lipid bilayer. Moreover, we used 10 and 20 times lower concentrations of Me-β-CD and TM-β-CD as compared with Hp-β-CD to achieve complete mTHPC solubilization during DCL preparation. Therefore, in the case of Hp-β-CD we have the large extent of free β-CD molecules, leading to the quick HDCL1 destabilization compared to MDCL1 and TDCL1.

In fine, DCL is rather stable nanoconstruct, which contains mTHPC mainly in inclusion complexes in the inner aqueous core of liposomes. It means that mTHPC delivery by DCLs should be quite different from conventional liposomes (Foslip^®^) and it may lead to the significant enhancement of mTHPC penetration in tumor tissue in vitro.

### 3.2. mTHPC Delivery to the Tumor Cells In Vitro

**Figure 4:**
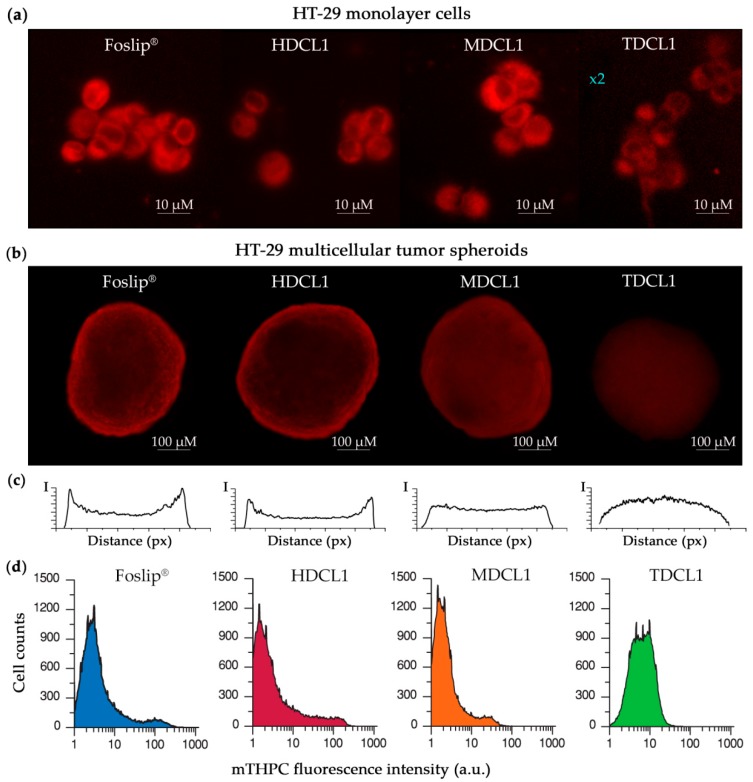
(**a**) Fluorescence images of mTHPC in different formulations in HT-29 monolayer cells 3 h post-incubation. (**b**) Fluorescence patterns of mTHPC in different formulations in HT-29 MCTSs 24 h post-incubation. (**c**) Linear profiles of mTHPC fluorescence intensity in HT-29 MTCSs treated by different mTHPC liposomal formulations for 24 h. (**d**) Histograms of mTHPC fluorescence in function of cell counts in HT29 spheroids after 24 h incubation with different mTHPC formulations. Log scale mTHPC fluorescence is shown on the x-axis. mTHPC concentration for all samples was 4.5 µM.

As stated above, the main purpose for encapsulating of mTHPC in the form of inclusion complexes in liposomes is to control PS release by β-CDs after liposome destruction in the medium. In order to investigate the validity of this hypothesis, we analyzed intracellular localization of mTHPC in HT29 human colon adenocarcinoma monolayer cells and PS distribution in HT29 MCTSs after pre-treatment with DCLs (Figure 4). Preliminary studies have shown that cellular uptake strongly depends on the absolute mTHPC concentration loaded in DCLs (data not shown). We demonstrated that DCLs containing less mTHPC (DCL2 and DCL3) exhibited lower accumulation in HT29 cells when compared with DCL1. Therefore, for our further in vitro studies we selected only HDCL1, MDCL1 and TDCL1.

Figure 4a displays the fluorescence images of HT29 cells treated with mTHPC and Foslip^®^ for 3 h. All formulations deliver mTHPC into the cells and we did not observe an obvious difference in intracellular localization between Foslip^®^ and DCLs. mTHPC is predominantly localized in the perinuclear region with diffuse fluorescence in the cytoplasm, as consistent with literature data related to Foslip^®^ [37]. It is worth to note that fluorescence intensity of TDCL1 in HT29 cells is significantly lower compared with other mTHPC formulations, perhaps because of the tight binding of mTHPC in TM-β-CD complexes.

Understanding the PS distribution processes in target tissues is a primary factor that is responsible for prediction of antitumor efficiency of photodynamic agents. MCTSs represent avascular regions found in many solid tumor tissues and allow for simulating the penetration and intratumor transport of anticancer nanomedicines, including photoactive NPs [38,39]. In the present work, HT-29 MCTSs were generated by spinner technique and filtered by the size from 380 to 520 µM for further experiments. Figure 4b displays epifluorescence imaging of the intact spheroids treated by Foslip^®^ and mTHPC-DCLs for 24 h. As seen on these images, the red fluorescence pattern of mTHPC was mainly localized on the periphery of Foslip^®^-treated spheroids, consistent with the literature data [40]. It is worth noting that HT-29 MCTSs that were treated with free mTHPC demonstrated similar peripherical profiles of fluorescence distribution [9]. The corresponding linear profile of mTHPC fluorescence across MTCSs, as presented in Figure 4c, demonstrates the presence of intensity peaks in the outer rim of spheroids that were treated with Foslip^®^. MCTSs exposed to HDCL1 also display high fluorescence in the outer rim of spheroids. The significant changes of mTHPC distribution in HT-29 MCTSs were observed for DCLs that contained mTHPC inclusion complexes with methylated β-CDs (MDCL1 and TDCL1). In the case of MDCL1, the intensity peaks on the periphery were smoothed, displaying an increase in the penetration depth of mTHPC. Similar changes of mTHPC distribution in spheroids were reported for free mTHPC/CD complexes in our recent paper [9]. However, the strongest changes of the mTHPC fluorescence pattern in MCTSs were observed for TDCL1. Linear profiles obtained after incubation of MCTSs with TDCL1 display spherical pattern with the maximal fluorescence intensity in the center. Taking into the account distortions due to the limited penetration of excitation light and spherical geometry of spheroids one can conclude that such fluorescence pattern corresponds to the almost complete penetration of mTHPC in spheroid depth and probably homogeneous PS distribution between cells of spheroids. 

To confirm the DCLs-induced alterations of mTHPC distribution in spheroids we used a flow cytometry technique (Figure 4d). The spheroids treated with various mTHPC formulations were trypsinized after 24 h incubation and analyzed by flow cytometry to assess the heterogeneity of mTHPC distribution between spheroid cells. Foslip^®^-treated spheroids exhibited a strong heterogeneity of mTHPC distribution between the cells. The distribution is broad and consists of several peaks. Obviously, mTHPC accumulates insufficiently in the deep layers of spheroids. Application of HDCL1 for mTHPC delivery in MCTSs does not significantly affect its distribution. Meanwhile, in the case of MDCL1, the distribution histogram slightly changes, still conserving strong heterogeneity similarly to Foslip^®^ and HDCL1. Finally, the incubation of MCTSs with TDCL1 results in an almost homogeneous distribution of mTHPC, supposing similar PS bioavailability for peripherical cells as well as for cells in the core of spheroid.

Thus, our data clearly demonstrate that DCLs significantly alter mTHPC distribution in spheroids. The alteration strength strongly depends on the type of used β-CD and increases in function of the affinity of β-CD to mTHPC in the following order (HDCL1 < MDCL1 < TDCL1). Taking into the account the influence of free CDs on mTHPC distribution in spheroids [9], one can suppose that the higher affinity of β-CD to mTHPC leads to the longer life-time of complex and as a matter of fact to the deeper delivery of mTHPC in the tumor tissue after the destruction of liposomes. In turn, deeper PS penetration results in its more homogeneous distribution of between cells [9]. Thus, in the case of TM-β-CD, the affinity is so high (>10^7^ M^−1^) [10] that mTHPC remains in the complex, even in the deep cell layers, resulting in almost homogeneous PS distribution in spheroid. 

## 4. Conclusions

Our study clearly demonstrated the potential use of drug-in-cyclodextrin-in-liposome formulation as a nanosized delivery system for mTHPC. The novel drug-loading procedure ensures the stable and efficient encapsulation of mTHPC bound to β-CDs into conventional liposomes. DCLs with various compositions were prepared in order to select the optimal nanoconstruct for mTHPC delivery in the target tissues. This study shows that liposomes may be effectively used as a reservoir for mTHPC/β-CDs complexes. It was demonstrated that TM-β-CD-based DCL retains almost all mTHPC in inclusion complexes and it remains stable for more than three months. Moreover, TDCL-treated tumor spheroids homogenously accumulated mTHPC across spheroid volume supposing optimal PS distribution in tumor tissue.

In long term perspectives, the limited accumulation of mTHPC delivered by DCLs should be optimized additionally. We suppose that double loading of drug could be applied to increase the total amount of mTHPC in DCLs and, perhaps, improving cellular uptake of TDCL. Currently, we are investigating biopharmaceutical properties of double loaded mTHPC-TDCL using in vitro 3D tumor models.

## Figures and Tables

**Table 1:** Composition of the different mTHPC-DCL formulations.

Formulation	CD	CD (mM)	mTHPC (mM)	Lipid (mM)
**Empty liposomes**	**-**	-	-	26
**DCLs with Hp-β-CD ^1^**				
HDCL 1	Hp-β-CD	200	5	26
HDCL 2			1.7	
HDCL 3			0.5	
**DCLs with Me-β-CD ^2^**				
MDCL 1	Me-β-CD	20	5	26
MDCL 2			1.7	
MDCL 3			0.5	
**DCLs with TM-β-CD ^3^**				
TDCL 1	TM-β-CD	10	5	26
TDCL 2			1.7	
TDCL 3			0.5	

^1^ Lipid vesicles with mTHPC/Hp-β-CD complexes encapsulated into the inner core. ^2^ Lipid vesicles with mTHPC/Me-β-CD complexes encapsulated into the inner core. ^3^ Lipid vesicles with mTHPC/TM-β-CD complexes encapsulated into the inner core.

**Table 2:** Characterization parameters of different liposomal formulations of mTHPC.

Formulation	EE (%)	Size (nm)	Polydispersity Index	Zeta-Potential (mV)
Foslip^®^	>85 [16]	113.6 ± 0.7	0.110 ± 0.015	−34.4 ± 4.3
**DCLs with Hp-β-CD**				
HDCL 1	7	137.7 ± 3.3	0.067 ± 0.030	−38.1 ± 1.9
HDCL 2	13	128.5 ± 0.3	0.037 ± 0.015	−36.7 ± 0.8
HDCL 3	17	125.7 ± 0.9	0.050 ± 0.004	−37.3 ± 1.6
**DCLs with Me-β-CD**				
MDCL 1	5	132.7 ± 0.8	0.045 ± 0.012	−37.8 ± 1.8
MDCL 2	7	141.0 ± 2.2	0.101 ± 0.019	−39.0 ± 2.6
MDCL 3	9	142.2 ± 0.8	0.073 ± 0.022	−36.4 ± 0.9
**DCLs with TM-β-CD**				
TDCL 1	7	135.9 ± 1.4	0.101 ± 0.034	−38.1 ± 1.2
TDCL 2	9	139.2 ± 0.9	0.065 ± 0.017	−36.9 ± 1.5
TDCL 3	14	130.6 ± 1.3	0.078 ± 0.026	−37.3 ± 2.1
